# Normative and Maladaptive Personality Trait Models of Mood, Psychotic, and Substance Use Disorders

**DOI:** 10.1007/s10862-018-9688-0

**Published:** 2018-06-13

**Authors:** Laura M. Heath, Lauren Drvaric, Christian S. Hendershot, Lena C. Quilty, R. Michael Bagby

**Affiliations:** 1 Department of Psychology, University of Toronto, 1265 Military Trail, Toronto, ON M1C 1A4 Canada; 2 Institute of Medical Science, University of Toronto, Toronto, ON Canada; 3 Campbell Family Mental Health Research Institute, Centre for Addiction and Mental Health, Toronto, ON Canada; 4 Department of Psychiatry, University of Toronto, Toronto, ON Canada

**Keywords:** Five Factor Model, NEO, PID-5, Psychopathology, *DSM-5*

## Abstract

The Personality Inventory for *DSM-5* (PID-5) is a questionnaire developed to assess the five domains represented in the alternative model for personality disorders proposed in Section III of the *DSM-5*. This study examined the ability of the PID-5 to distinguish between different mental disorders compared to a questionnaire measure of the five-factor model (FFM) of normative personality. The study included the administration of the PID-5 and Revised NEO Personality Inventory (NEO PI-R), a measure of the FFM, to treatment-seeking individuals with Depressive, Bipolar, Psychotic, and Alcohol Use Disorders (AUD). Diagnostic groups were compared at the domain level of PID-5 and NEO PI-R, with sex and age as covariates. The main findings on the PID-5 included higher Detachment scores for Bipolar and Depressive Disorders than Psychotic and AUDs, lower Psychoticism/higher Disinhibition scores for the AUD group compared to all other groups, and lower Negative Affect for the Psychotic Disorders versus AUD group. On the NEO PI-R, the AUD diagnostic group was associated with lower Conscientiousness and Agreeableness scores compared to all other groups, and lower Neuroticism scores than the Bipolar and Depressive groups. Group pairwise comparisons did not appear to show many differences between the PID-5 and NEO PI-R. The results suggest that the alternative *DSM-5* model for personality disorders may have clinical utility in distinguishing personality profiles between diagnostic groups. These findings emphasize the importance of additional research on the capacity of maladaptive personality to contribute to the assessment of differential diagnoses.

**Electronic supplementary material:**

The online version of this article (10.1007/s10862-018-9688-0) contains supplementary material, which is available to authorized users.

## Introduction

The five-factor model (FFM) of personality has been widely accepted as a comprehensive model of normal range personality traits, comprising five domains: Neuroticism, Extraversion, Openness, Agreeableness, and Conscientiousness (McCrae and John 1992). The FFM was first developed using a lexical approach to examine trait terms within existing languages, which converged well onto a five-factor structure (Widiger and Trull 2007). A plethora of research has examined the role of the FFM in psychopathology. Despite efforts to identify personality traits associated with different mental disorders, the domains of the FFM have been criticized for being too broad to have any diagnostic utility (Clark 1993). Literature reviews on the associations between the FFM personality domains and varying mental disorders (i.e., anxiety, depressive, substance use, and personality disorders) demonstrated high Neuroticism and low Conscientiousness across all diagnostic groups (see meta-analyses: Kotov et al. 2010; Samuel and Widiger 2008). The lack of psychopathological content in the FFM may be contributing to the general, non-specific personality profile characteristic of all mental disorders using this model of normal range personality.

The alternative model for personality disorders (PDs) proposed in Section III of the *DSM-5* (American Psychiatric Association 2013) incorporates maladaptive personality traits that were derived from literature reviews and *DSM-5* workgroup deliberations, followed by iterative analyses to capture the maladaptive trait features of *DSM-IV-TR* PDs (Krueger et al. 2012). The Personality Inventory for *DSM-5* (PID-5; Krueger et al. 2012) measures the five domains of maladaptive personality in the alternative model: Negative Affect, Detachment, Psychoticism, Antagonism, and Disinhibition, which partially correspond with the pathological “poles” of the FFM personality domains (Skodol et al. 2015). Four of the five domains have been shown to correlate with four of the domains of the FFM. Negative Affect correlates positively with Neuroticism, whereas Detachment correlates negatively with Extraversion, Antagonism with Agreeableness, and Disinhibition with Conscientiousness (Krueger et al. 2012; Watson et al. 2013; for review see Al-Dajani et al. 2016). The relationship between Psychoticism and Openness is more complex and requires an understanding of the different conceptualizations of Openness (Chmielewski et al. 2014; Suzuki et al. 2017).

Section III of the *DSM-5* proposes that elevation of pathological personality traits, as measured by the PID-5, contribute to clinical decisions about degree of disability, treatment type and intensity, and prognosis (APA 2013). Since it is unlikely that most clinical contexts would have the capacity to administer multiple comprehensive measures of personality, it would be efficient to use the PID-5 to support the diagnosis of personality disorders as well as other clinical decisions. Therefore, it is important to research this personality trait system to aid in our understanding of the clinical utility of the instrument and the alternative *DSM-5* model. Recent research has begun to examine the relationships between maladaptive personality traits and mental disorders. For example, Carlotta et al. (2015) found that PID-5 domains of Detachment and Antagonism differentiated high- from low-risk gamblers. To our knowledge, this was the first study to use the PID-5 to examine maladaptive personality traits in individuals at risk for an Axis I disorder. These findings suggest that the *DSM-5* model of dimensional personality pathology may be able to differentiate maladaptive from normative behaviours.

The association of Detachment and Antagonism with gambling risk may be generalized to a broader set of externalizing disorders. A recent study by Sleep et al. (2017) assessed the relationships between PID-5 and FFM personality domains and externalizing and internalizing behaviours among an undergraduate sample. Using multivariate regression, Disinhibition, Antagonism, and low Detachment had significant unique associations with alcohol and drug misuse, as did their FFM counterparts of low Conscientiousness, low Agreeableness, and Extraversion. In contrast, Negative Affect and Detachment both had significant associations with past-week experiences of anxiety and depression, while only Neuroticism had a significant unique effect from the FFM. However, no studies to date have assessed whether the PID-5 can identify personality distinctions between different mental disorders in a clinical sample.

The present study was an initial attempt to examine the discriminant validity of the PID-5 to distinguish between Psychotic, Bipolar, Depressive, and Alcohol Use Disorders (AUD). These analyses were also conducted with the FFM, as measured using the Revised NEO Personality Inventory (NEO PI-R; Costa and McCrae 1992), with the expectation to replicate previous findings in the literature.

Table 1 displays the hypotheses of differences in PID-5 and NEO PI-R scores between the mental disorders. No hypotheses were made for the domains of Negative Affect/Neuroticism since high Neuroticism is a non-specific vulnerability factor for psychopathology (Hink et al. 2013; Malouff et al. 2005; Widiger and Oltmanns 2017).

**Table 1:** Hypotheses of PID-5 and NEO PI-R score differences between mental disorders

PID-5/NEO PI-R Domain	Group Differences	Hypotheses	Rationale
Negative Affect/ Neuroticism		No hypothesized group differences	High neuroticism has been shown to be a non-specific vulnerability factor for psychopathology (Hink et al. 2013; Malouff et al. 2005; Widiger and Oltmanns 2017).
Detachment/ Extraversion	B-D	Bipolar Disorders lower Detachment/higher Extraversion compared to Depressive Disorders	Quilty et al. (2013b) demonstrated that higher Extraversion (and Agreeableness) predicted bipolar disorder versus unipolar depression. Tackett et al. (2008) showed that individuals with Bipolar Disorder scored higher on Extraversion than those with other internalizing disorders.
	B-P B-A D-P D-A	Bipolar and Depressive Disorders higher on Detachment/lower Extraversion than the Psychotic and AUD groups	Meta-analyses that mood disorders have lower Extraversion than other disorders (Kotov et al. 2010; Malouff et al. 2005)
Psychoticism/ Openness	B-P D-P P-A	Psychotic Disorders higher Psychoticism than all other diagnostic groups No differences on Openness	Lack of consistent findings for the role of Openness in psychotic disorders (Dinzeo and Docherty 2007)
Antagonism/ Agreeableness	B-D	Bipolar lower on Antagonism/higher Agreeableness compared to Depressive Disorders	Quilty et al. (2013b) demonstrated that higher Agreeableness (and Extraversion) predicted bipolar disorder versus unipolar depression.
	B-P	No difference	In an examination of personality differences between patients with remitted unipolar depression, euthymic bipolar disorder, and residual schizophrenia, only patients with depression had significantly higher Agreeableness than the schizophrenia patients (Bagby et al. 1997).
	D-P	Depressive lower Antagonism/higher Agreeableness than Psychotic Disorders	
	B-A D-A P-A	AUD higher Antagonism/lower Agreeableness than all other diagnostic groups	It has been well established that higher disinhibition and lower Agreeableness are characteristic of substance use disorders more so than other disorders (Hopwood et al. 2007; Sleep et al. 2017; Terracciano et al. 2008).
Disinhibition/ Conscientiousness	B-A D-A P-A	AUD higher Disinhibition/lower Conscientiousness than all other disorders	

Group differences are denoted as follows: B-D –Bipolar vs. Depressive; B-P – Bipolar vs. Psychotic; B-A – Bipolar vs. AUD; D-P– Depressive vs. Psychotic; D-A- Depressive vs. AUD; P-A – Psychotic vs. AUD

## Methods

### Participants

Data were collected from study protocols that took place at the Centre for Addiction and Mental Health (CAMH). The diagnostic groups of Bipolar and Related Disorders (*n* = 22), Depressive Disorders (*n* = 30), and Schizophrenia Spectrum and Other Psychotic Related Disorders (*n* = 78) were recruited at CAMH as part of the *DSM-5* Field Trials (Clarke et al. 2013; Narrow et al. 2013; Regier et al. 2013). Prior to the first trial visit, the referring/treating clinician diagnosed participants entering treatment at CAMH based on case file review and clinical impression. Following participation in the *DSM-5* Field Trials, participants completed additional measures including those detailed below. This sample has been examined previously by Quilty et al. (2013a) in a report about the psychometric properties of the PID-5 and NEO PI-R on the full sample of participants from the *DSM-5* Field Trials, including additional diagnostic groups such as personality disorders that were not assessed in the current study. The present analyses also differ from previous work by assessing the effects of diagnostic category on the PID-5 and NEO PI-R scores.

The present study collapsed schizophrenia (*n* = 45) and schizoaffective disorders (*n* = 30) into a Psychotic Disorders group because there were no significant differences in demographic factors or domain scores between the two groups. The Psychotic Disorders group had a mean age of 42.60 ± 12.28 and 44% were females. The study sample also included patients who were classified with Bipolar or Depressive Disorder. The mean age of the Bipolar and Related Disorders group was 40.59 ± 13.98 and 55% were females, and the Depressive Disorders group had a mean age of 45.27 ± 15.38, and 50% were female. The majority of the sample from the *DSM-5* Field Trials were Caucasian (66%), 10% identified as Asian, 8% identified as Black – Caribbean (3%), African (3%), or North American (2%), 4% were of mixed racial identity, two people (1.6%) each identified as Aboriginal, Indian Caribbean, Latin American, and Middle Eastern, 4% identified as Other, and 2% preferred not to answer.

The AUD group (*n* = 28) consisted of patients presenting for treatment for alcohol use disorder at the same clinical site. Participants were enrolled in a separate research protocol, during which the NEO-PI-R and PID-5 were administered for purposes of generating an AUD reference group for the present analyses. Participants met criteria for *DSM-IV* alcohol dependence based on a Structured Clinical Interview for *DSM-IV* Axis I Disorders (SCID-I) and had no concurrent psychosis or drug dependence. The mean age of the AUD group was 43.41 ± 9.45 and 30.6% were females. Twenty-three of the participants identified as Caucasian (82%), two (7%) identified as Aboriginal, one person (4%) identified as Asian, and two (7%) had missing data. The clinicians who referred the patients to the *DSM-5* Field Trial studies used the primary diagnosis, as conveyed by the referring clinicians. Information on comorbidities was not available.

### Measures

**Revised NEO Personality Inventory** (NEO PI-R; Costa and McCrae 1992). The NEO PI-R is a 240-item self-report questionnaire designed to capture the five domains of the FFM: Neuroticism, Extraversion, Openness, Conscientiousness, and Agreeableness. Each domain encompasses six facet scales and items are rated on a scale of 0–4 (0 = strongly disagree to 4 = strongly agree). The present study demonstrated internal consistencies for the NEO PI-R from α = 0.70 (Openness) to 0.86 (Neuroticism). The raw scores were converted to T-scores^1^ (mean 50; SD 10) from gendered American normative samples (Costa and McCrae 1992).

**Personality Inventory for DSM-5** (PID-5; Krueger et al. 2013). The PID-5 is a 220-item self-report questionnaire aimed to assess maladaptive personality characteristics corresponding to the *DSM-5* Section III model of personality psychopathology (APA 2013). The inventory comprises 25 facet scales that delineate 5 higher-order domains: Negative Affect, Detachment, Antagonism, Disinhibition, and Psychoticism. Items are rated on a 4-point Likert scale (0 = very false or often false to 3 = very true or often true). The PID-5 has shown adequate psychometric properties, including internal consistency, test-retest reliability, and construct validity (for a review, see Al-Dajani et al. 2016). Research has also supported the convergent and discriminant validity for the PID-5 scales compared to other measures of maladaptive personality traits (Crego and Widiger 2016). The psychometric properties of the PID-5 and NEO PI-R for some of the sample have been reported previously by Quilty et al. (2013a). The Cronbach α values for the domains in the present study ranged from 0.71 (Disinhibition) to 0.87 (Detachment). While there are a number of different ways to score the PID-5, we used the initial derivation from Kreuger et al. (2012), whereby domain scores were computed using the mean of the facet scores loading onto each domain. Each domain scale score can range from 0 to 3.

**Structured Clinical Interview for** ***DSM-IV*** **Axis I Disorders** (SCID-I; First et al. 1996). The SCID-I is a structured interview assessing *DSM-IV* Axis I disorders that has demonstrated a high level of reliability and validity (First and Gibbon 2004). The SCID-I was used to assess alcohol dependence. All participants comprising the AUD group fulfilled DSM-IV criteria for dependence.

### Statistical analyses

One-way multivariate analyses of covariance (MANCOVAs) were performed to assess the relationship between diagnostic group and NEO PI-R and PID-5 domain scores, with sex and age as covariates. All analyses were performed with SPSS version 24 (IBM Corp.). Levene’s test of equality of error variances was not significant for the PID-5 or NEO PI-R MANCOVAs, demonstrating that the error variance of the dependent variable is equal across groups. The alpha levels of pairwise comparisons were corrected for multiple comparisons using Bonferroni method for each instrument (i.e., 6 group comparisons × 5 domains = 30 tests; adjusted *α* = 0.002) and statements of significance were based on these adjusted values. Results are described as the mean value and 95% confidence intervals [lower bound, upper bound]. Cohen’s *d* effect sizes can be interpreted as small (*d* = 0.2), medium (*d* = 0.5), and large (*d* = 0.8), according to benchmarks suggested by Cohen (1988).

Supplementary material is provided to examine diagnostic group differences at the facet level. Statistical analyses of group differences at the facet level are limited by the lack of statistical power required to control for family-wise error. Therefore, supplementary Tables 1a and 1b demonstrate the estimated mean facet scores and the effect sizes of pairwise comparisons, with covariates of sex and age, of all diagnostic groups for the PID-5 and NEO PI-R, respectively. Supplementary Figs. 1 and 2 portray the effect sizes of each facet score for the PID-5 and NEO PI-R, respectively, collapsed across pairwise comparisons.

## Results

Multivariate tests found a significant main effect of age on PID-5, *F*(5,148) = 2.63, *p* = 0.026, partial η^2^ = 0.08 and NEO PI-R domain scores, *F*(5,154) = 3.28, *p* = 0.008, partial η^2^ = 0.10. There was also a significant main effect of sex on NEO PI-R scores, *F*(5,154) = 2.49, *p* = 0.034, partial η^2^ = 0.08. Therefore, sex and age were entered as covariates in all subsequent analyses.^2^ Refer to Table 2 for the estimated marginal means of the domains by diagnostic group and the effect sizes of the pairwise comparisons.

**Table 2:** Patient Group Comparisons for the PID-5 and NEO PI-R personality domains (*n* = 158)

	Bipolar *n* = 22		Depressive *n* = 30		Psychotic *n* = 78		AUD^a^ *n* = 28		Cohen’s *d*^b^					
	*M*	*95% CI*	*M*	*95% CI*	*M*	*95% CI*	*M*	*95% CI*	*B-D*	*B-P*	*B-A*	*D-P*	*D-A*	*P-A*
PID-5 Domains^c^														
Negative Affect	1.19	[1.01, 1.37]	1.22	[1.07, 1.38]	0.98	[0.89, 1.08]	1.32	[1.16, 1.48]	0.07	0.50	0.31	0.57	0.24	**0.81**
Detachment	1.45	[1.27, 1.64]	1.48	[1.31, 1.64]	1.16	[1.06, 1.26]	0.92	[0.75, 1.09]	0.07	**0.64**	**1.18**	**0.71**	**1.24**	0.53
Psychoticism	1.24	[1.05, 1.44]	1.23	[1.06, 1.40]	1.07	[0.96, 1.17]	0.76	[0.58, 0.93]	0.02	0.36	**1.01**	0.34	**0.99**	**0.65**
Antagonism	1.02	[0.84, 1.21]	0.99	[0.83, 1.14]	0.87	[0.77, 0.96]	0.76	[0.60, 0.93]	0.07	0.34	0.59	0.28	0.53	0.25
Disinhibition	1.09	[0.95, 1.23]	1.05	[0.93, 1.17]	1.00	[0.93, 1.08]	1.48	[1.35, 1.60]	0.12	0.27	**1.18**	0.15	**1.30**	**1.43**
NEO PI-R Domains^d^														
Neuroticism	73.04	[67.97, 78.11]	72.84	[68.50, 77.18]	68.54	[65.84, 71.23]	63.80	[59.66, 67.94]	0.02	0.37	**0.80**	0.36	**0.78**	0.41
Extraversion	54.70	[49.64, 59.75]	48.51	[44.18, 52.84]	53.73	[51.04, 56.42]	52.77	[48.64, 56.90]	0.52	0.08	0.17	0.43	0.37	0.08
Openness	64.80	[59.90, 69.69]	61.91	[57.71, 66.10]	61.00	[58.40, 63.61]	58.49	[54.49, 62.48]	0.25	0.33	0.56	0.08	0.31	0.22
Agreeableness	56.87	[51.19, 62.56]	55.34	[50.47, 60.21]	55.66	[52.64, 58.69]	46.17	[41.52, 50.81]	0.11	0.09	**0.82**	0.02	**0.71**	**0.73**
Conscientiousness	56.85	[51.28, 62.42]	52.54	[47.77, 57.31]	54.10	[51.14, 57.06]	39.49	[34.94, 44.04]	0.33	0.21	**1.35**	0.12	**1.02**	**1.14**

Values represent the estimated marginal means and 95% confidence interval [lower bound, upper bound] with covariates of sex and age = 42.99 in the model

^a^Alcohol use disorder

^b^Effect sizes are represented as, B-D – Bipolar to Depressive; B-P – Bipolar to Psychotic; B-A – Bipolar to AUD; D-P – Depressive to Psychotic; D-A - Depressive to AUD; P-A – Psychotic to AUD

Significant pairwise comparisons (Bonferroni adjusted *α* < 0.002) are **boldfaced**

^c^PID-5 items are scored on a 4-point Likert scale ranging from 0 to 3

^d^NEO-PI-R domain *T*-scores from gendered American normative samples (Costa and McCrae 1992) are presented

### PID-5

In the test of between-subjects effects, significant main effects of *DSM-5* diagnostic groups were found on all PID-5 domains except for Antagonism: Negative Affect *F*(3, 152) = 5.47, *p* = .001, partial η^2^ = 0.10, Detachment *F*(3, 152) = 9.79, *p* = .0001, partial η^2^ = 0.16, Psychoticism *F*(3, 152) = 6.27, *p* = .0001, partial η^2^ = 0.11, and Disinhibition *F*(3, 152) = 14.37, *p* = .0001, partial η^2^ = 0.22.

### PID-5 Pairwise Comparisons

#### Negative Affect

Individuals with AUD scored significantly higher on Negative Affect compared to the Psychotic Disorders group.

#### Detachment

Bipolar and Depressive groups scored significantly higher on Detachment than the Psychotic and AUD groups.

#### Psychoticism

Individuals with AUD scored lower on Psychoticism compared to all other diagnostic groups.

#### Antagonism

No significant group differences were found on Antagonism.

#### Disinhibition

Individuals with AUD were significantly higher on Disinhibition compared to all other diagnostic groups.

### NEO PI-R

Tests of between-subject effects for the NEO PI-R domains demonstrated significant effects of diagnostic group for Neuroticism *F*(3, 158) = 3.91, *p* = 0.01, partial η^2^ = 0.07, Agreeableness *F*(3, 158) = 4.55, *p* = 0.004, partial η^2^ = 0.08, and Conscientiousness *F*(3, 158) = 11.38, *p* = 0.0001, partial η^2^ = 0.18, no significant effects were found for Extraversion or Openness.

### NEO PI-R Pairwise Comparisons

#### Neuroticism

There were significantly lower scores on Neuroticism for the AUD group compared to both Bipolar and Depressive groups.

#### Extraversion

There were no significant differences between mental disorders on Extraversion.

#### Openness

There were no significant group differences on Openness.

#### Agreeableness

The AUD group scored significantly lower on Agreeableness compared to all other diagnostic groups.

#### Conscientiousness

The AUD group also scored significantly lower on Conscientiousness compared to all other diagnostic groups.

### PID-5 vs. NEO PI-R

Table 2 was examined to compare the two models in distinguishing between the diagnostic groups. Although the PID-5 demonstrated a greater number of significant pairwise comparisons, the overall results are quite similar. The findings suggest that there are no major differences between the PID-5 and NEO PI-R, with both demonstrating particularly notable personality distinctions between individuals with AUD and all other diagnostic groups.

## Discussion

The present study investigated differences in normal and maladaptive personality traits between Bipolar, Depressive, Psychotic, and AUDs using the PID-5 and NEO PI-R. It was hypothesized that Neuroticism/Negative Affect would not show group differences because this trait is characteristic of all mental disorders. However, Neuroticism was significantly lower among individuals seeking treatment for AUD than those with Bipolar or Depressive Disorder. Nevertheless, all groups were elevated compared to the American normative samples, scoring at least 1 SD above the norm.

Contrary to the hypotheses and results from Quilty et al. (2013b), neither the FFM nor the alternative model of PDs differentiated Bipolar and Depressive Disorders on any personality domain. An earlier study found no differences in FFM domains between depressive and bipolar groups, so these results are not unfounded (Bagby et al. 1997). Other research has found that there can be significant differences between diagnostic groups at the facet level of personality that may be lost in the assessment of higher order domains (Quilty et al. 2013b; Rector et al. 2012). Supplementary Tables 1a and 1b further showed no medium or large effect sizes for comparisons between Depressive and Bipolar Disorders at the facet level. Another explanation for the lack of group differences between Bipolar and Depressive Disorders in the present study could be due to the method of unstructured diagnostic assessment that was employed through chart review.

Results did support the hypothesis that a primary diagnosis of either Bipolar or Depressive Disorder would be associated with significantly higher scores on Detachment compared to Psychotic Disorders and AUD, but not the correlated FFM domain of Extraversion. Both Extraversion and Openness had no significant main effects of mental disorder classification, whereas the related PID-5 domains had significant pairwise comparisons. The distinction between groups on the PID-5 domain of Psychoticism but not the NEO PI-R Openness adds to the controversial literature on the limitations of this domain and its associations with *DSM-5* Schizophrenia Spectrum and Other Psychotic Disorders (Chmielewski et al. 2014). However, the scores on Psychoticism by diagnostic group were not as predicted.

The Psychotic Disorders group scored significantly higher on Psychoticism than those with AUD, but so did individuals with Bipolar and Depressive Disorders. The unstructured interview method used to classify diagnostic groups may be responsible for these results, considering the difficulty in making a differential diagnosis between mood and psychotic disorders, particularly bipolar and schizoaffective disorder (Abrams et al. 2008; Benabarre et al. 2001; Marneros 2003). Furthermore, the stricter exclusion criteria of the study from which the AUD group was derived, particularly concurrent psychosis, may have contributed to the lower Psychoticism scores among those with AUD compared to the diagnostic groups from the *DSM-5* Field Trials.

In addition to scoring lower on Neuroticism and Detachment, individuals with AUD also had significantly lower Agreeableness, but not Antagonism, and higher Disinhibition/lower Conscientiousness than all other diagnostic groups. In a study by Trull and Sher (1994) in which major depression, anxiety, and substance use disorders were related to the FFM domains in a canonical analysis, a significant canonical variable representing a non-depressed substance abuse dimension was characterized by higher Extraversion, and lower Neuroticism, Agreeableness, and Conscientiousness. These findings were mostly replicated in the present study; there were no group differences on Extraversion, but the complementary PID-5 domain of Detachment was significant.

When examining the average effect sizes of pairwise comparisons collapsed across domains, the PID-5 and NEO PI-R do not demonstrate dramatic differences. Nevertheless, these results demonstrate the clinical utility of the *DSM-5* alternative model for PDs to differentiate between mental disorders. A limitation to the study is variation in diagnostic methodology across groups, particularly the use of chart review to diagnose participants entering the *DSM-5* Field Trials, whereas the diagnosis of alcohol dependence was based on the SCID-I. Additionally, only the primary diagnosis for each participant was used and comorbid disorders were not considered. Despite this limitation, there were strong differences in maladaptive personality based on the primary diagnoses. There was also not enough statistical power to conduct analyses of lower order facet differences. Further research is required to replicate findings and provide an empirical basis for the adoption of a dimensional model of maladaptive personality in the diagnostic assessment of mental disorders.

## Electronic supplementary material

ESM 1 (DOCX 50 kb)

ESM 2 (PDF 97 kb)

ESM 3 (PDF 107 kb)

## Footnotes

1 T-scores were used for all analyses with the NEO PI-R to examine comparison to a normative sample, however the results did not differ from the raw scores

2 Without covariates of age and sex for the MANOVAs of PID-5 and NEO PI-R scores by diagnostic groups, the significance levels changed, but the pattern of results and the effect sizes were consistent.
