# Correction to: Normative and Maladaptive Personality Trait Models of Mood, Psychotic, and Substance Use Disorders

**DOI:** 10.1007/s10862-018-9693-3

**Published:** 2018-08-23

**Authors:** Laura M. Heath, Lauren Drvaric, Christian S. Hendershot, Lena C. Quilty, R. Michael Bagby

**Affiliations:** 1 Department of Psychology, University of Toronto, 1265 Military, Trail, Toronto, ON M1C 1A4 Canada; 2 Institute of Medical Science, University of Toronto, Toronto, ON Canada; 3 Campbell Family Mental Health Research Institute, Centre for Addiction and Mental Health, Toronto, ON Canada; 4 Department of Psychiatry, University of Toronto, Toronto, ON Canada

**Correction to: J. Psychopathol. Behav. Assess. (2018)**

10.1007/s10862-018-9688-0

The article Normative and maladaptive personality trait models of mood, psychotic, and substance use disorders, written by Laura M. Heath, Lauren Drvaric, Christian S. Hendershot, Lena C. Quilty, and R. Michael Bagby was originally published electronically on the publisher’s internet portal (currently SpringerLink) on June 13, 2018 without open access.

With the author (s)’ decision to opt for Open Choice the copyright of the article changed on August 22, 2018 to © The Author (s) 2018 and the article is forthwith distributed under the terms of the Creative Commons Attribution 4.0 International License (http://creativecommons.org/licenses/by/4.0/), which permits use, duplication, adaptation, distribution and reproduction in any medium or format, as long as you give appropriate credit to the original author (s) and the source, provide a link to the Creative Commons license and indicate if changes were made.

The original article has been corrected.

**Open Access** This article is distributed under the terms of the Creative Commons Attribution 4.0 International License (http://creativecommons.org/licenses/by/4.0/), which permits use, duplication, adaptation, distribution and reproduction in any medium or format, as long as you give appropriate credit to the original author (s) and the source, provide a link to the Creative Commons license and indicate if changes were made.

