# Supplementary material for: Normative and Maladaptive Personality Trait Models of Mood, Psychotic, and Substance Use Disorders
Source: J Psychopathol Behav Assess. 2018 Jun 13;40(4):606–13. doi: 10.1007/s10862-018-9688-0 (PMC6223804; doi:10.1007/s10862-018-9688-0)
Supplement: Supplementary file 1 — (DOCX 50 kb) [file 10862_2018_9688_MOESM1_ESM.docx]

|  | Bipolar  *n* = 22 | | Depressive  *n* = 30 | | Psychotic  *n* = 78 | | AUD^a^  *n* = 28 | | Cohen’s *d*^b^ | | |  |  |  |
| --- | --- | --- | --- | --- | --- | --- | --- | --- | --- | --- | --- | --- | --- | --- |
|  |  |  |  |  |  |  |  |  |  |  |  |  |  |  |
|  | ***M*** | ***95% CI*** | ***M*** | ***95% CI*** | ***M*** | ***95% CI*** | ***M*** | ***95% CI*** | ***B-D*** | ***B-P*** | ***B-A*** | ***D-P*** | ***D-A*** | ***P-A*** |
| PID-5 Facets |  |  |  |  |  |  |  |  |  |  |  |  |  |  |
| Anxiousness | 0.99 | [0.81, 1.18] | 0.87 | [0.71, 1.03] | 0.91 | [0.81, 1.01] | 1.61 | [1.44, 1.77] | 0.43 | 0.24 | **1.09** | 0.13 | **1.32** | **1.18** |
| Emotional lability | 1.46 | [1.22, 1.71] | 1.51 | [1.29, 1.72] | 1.09 | [0.96, 1.22] | 1.26 | [1.04, 1.48] | 0.08 | **0.68** | 0.33 | **0.69** | 0.38 | 0.27 |
| Hostility | 1.09 | [0.86, 1.32] | 1.15 | [0.95, 1.35] | 0.84 | [0.72, 0.97] | 1.12 | [0.91, 1.32] | 0.09 | 0.47 | 0.05 | **0.54** | 0.05 | 0.49 |
| Perseveration | 1.22 | [0.99, 1.45] | 1.21 | [1.01, 1.41] | 1.04 | [0.92, 1.16] | 1.02 | [0.82, 1.23] | 0.02 | 0.35 | 0.35 | 0.32 | 0.32 | 0.04 |
| Restricted affectivity | 1.06 | [0.83, 1.28] | 1.15 | [0.96, 1.34] | 1.05 | [0.93, 1.17] | 0.92 | [0.72, 1.12] | 0.17 | 0.02 | 0.25 | 0.19 | 0.39 | 0.23 |
| Separation insecurity | 1.12 | [0.85, 1.38] | 1.27 | [1.03, 1.50] | 0.96 | [0.82, 1.11] | 0.95 | [0.71, 1.19] | 0.23 | 0.27 | 0.26 | 0.48 | 0.45 | 0.02 |
| Submissiveness | 1.20 | [0.92, 1.47] | 1.22 | [0.97, 1.46] | 1.03 | [0.88, 1.18] | 1.33 | [1.09, 1.58] | 0.03 | 0.27 | 0.19 | 0.27 | 0.15 | 0.44 |
| Anhedonia | 1.58 | [1.35, 1.82] | 1.66 | [1.46, 1.87] | 1.16 | [1.03, 1.28] | 1.19 | [0.98, 1.39] | 0.15 | **0.83** | **0.62** | **0.97** | **0.74** | 0.05 |
| Depressivity | 1.19 | [0.98, 1.39] | 1.29 | [1.11, 1.47] | 1.02 | [0.91, 1.13] | 0.93 | [0.74, 1.11] | 0.21 | 0.35 | **0.57** | **0.52** | **0.74** | 0.18 |
| Intimacy avoidance | 1.44 | [1.19, 1.69] | 1.47 | [1.26, 1.69] | 1.22 | [1.08, 1.35] | 0.56 | [0.34, 0.79] | 0.05 | 0.39 | **1.37** | 0.43 | **1.40** | **1.05** |
| Suspiciousness | 1.34 | [1.18, 1.49] | 1.27 | [1.14, 1.41] | 1.18 | [1.09, 1.26] | 0.92 | [0.78, 1.06] | 0.21 | **0.52** | **0.96** | 0.27 | **0.77** | **0.59** |
| Withdrawal | 1.34 | [1.12, 1.57] | 1.27 | [1.07, 1.47] | 1.07 | [0.95, 1.20] | 0.90 | [0.70, 1.10] | 0.14 | **0.55** | **0.78** | 0.38 | **0.62** | 0.30 |
| Attention seeking | 1.34 | [1.12, 1.56] | 1.20 | [1.02, 1.39] | 1.06 | [0.94, 1.17] | 1.36 | [1.17, 1.56] | 0.27 | **0.56** | 0.03 | 0.32 | 0.25 | 0.49 |
| Callousness | 0.81 | [0.65, 0.97] | 0.85 | [0.71, 0.99] | 0.66 | [0.57, 0.75] | 0.37 | [0.22, 0.51] | 0.09 | 0.36 | **1.02** | 0.45 | **1.10** | **0.70** |
| Deceitfulness | 1.08 | [0.86, 1.29] | 1.00 | [0.81, 1.19] | 0.94 | [0.82, 1.06] | 0.81 | [0.62, 1.01] | 0.14 | 0.28 | 0.45 | 0.11 | 0.31 | 0.24 |
| Grandiosity | 0.87 | [0.66, 1.06] | 0.83 | [0.67, 1.00] | 0.67 | [0.57, 0.78] | 0.59 | [0.42, 0.76] | 0.09 | 0.44 | **0.61** | 0.34 | **0.51** | 0.18 |
| Manipulativeness | 1.13 | [0.88, 1.38] | 1.09 | [0.87, 1.30] | 0.97 | [0.84, 1.11] | 1.00 | [0.77, 1.22] | 0.06 | 0.28 | 0.22 | 0.19 | 0.14 | 0.05 |
| Distractibility | 1.11 | [0.91, 1.31] | 1.08 | [0.90, 1.25] | 1.06 | [0.95, 1.17] | 1.39 | [1.21, 1.57] | 0.08 | 0.13 | 0.45 | 0.05 | **0.50** | **0.52** |
| Impulsivity | 1.25 | [1.02, 1.48] | 1.15 | [0.95, 1.35] | 1.21 | [1.09, 1.34] | 1.32 | [1.11, 1.52] | 0.20 | 0.08 | 0.11 | 0.12 | 0.29 | 0.18 |
| Irresponsibility | 0.89 | [0.71, 1.07] | 0.93 | [0.78, 1.09] | 0.74 | [0.65, 0.84] | 0.85 | [0.69, 1.01] | 0.11 | 0.38 | 0.07 | **0.53** | 0.15 | 0.21 |
| Rigid perfectionism | 0.98 | [0.76, 1.20] | 0.98 | [0.79, 1.18] | 0.84 | [0.72, 0.96] | 1.16 | [0.96, 1.36] | 0.00 | 0.29 | 0.31 | 0.28 | 0.30 | **0.54** |
| Risk taking | 0.96 | [0.84, 1.08] | 0.94 | [0.83, 1.04] | 0.87 | [0.81, 0.94] | 1.64 | [1.52, 1.74] | 0.06 | 0.31 | **1.85** | 0.26 | **2.00** | **2.33** |
| Eccentricity | 1.25 | [0.98, 1.51] | 1.21 | [0.98, 1.44] | 1.09 | [0.95, 1.24] | 0.98 | [0.75, 1.22] | 0.06 | 0.27 | 0.37 | 0.19 | 0.30 | 0.15 |
| Perceptual dysregulation | 0.99 | [0.80, 1.22] | 0.92 | [0.75, 1.08] | 0.83 | [0.73, 0.93] | 0.63 | [0.46, 0.80] | 0.17 | 0.38 | **0.78** | 0.21 | **0.61** | 0.41 |
| Unusual beliefs and experiences | 1.50 | [1.27, 1.73] | 1.54 | [1.34, 1.75] | 1.27 | [1.14, 1.39] | 0.58 | [0.37, 0.79] | 0.07 | 0.41 | **1.72** | 0.47 | **1.72** | **1.29** |

Supplementary Table 1a. Patient Group Comparisons for the PID-5 personality facets (*n* = 158)

Values represent the estimated marginal means and 95% confidence interval [lower bound, upper bound] with covariates of sex and age = 42.74 in the model.

^a^AUD – Alcohol use disorder

^b^Effect sizes are represented as, B-D –Bipolar to Depressive; B-P – Bipolar to Psychotic; B-A – Bipolar to AUD; D-P– Depressive to Psychotic; D-A- Depressive to AUD; P-A – Psychotic to AUD.

Effect sizes of medium magnitude (d = 0.5) or higher are **boldfaced**.

PID-5 items are scored on a 4-point Likert scale ranging from 0-3.

Supplementary Table 1b. Patient Group Comparisons for the NEO PI-R personality facets (*n* = 163)

|  | Bipolar  *n* = 22 | | Depressive  *n* = 30 | | Psychotic  *n* = 78 | | AUD^a^  *n* = 33 | | Cohen’s *d*^b^ | | |  |  |  |
| --- | --- | --- | --- | --- | --- | --- | --- | --- | --- | --- | --- | --- | --- | --- |
|  |  |  |  |  |  |  |  |  |  |  |  |  |  |  |
|  | ***M*** | ***95% CI*** | ***M*** | ***95% CI*** | ***M*** | ***95% CI*** | ***M*** | ***95% CI*** | ***B-D*** | ***B-P*** | ***B-A*** | ***D-P*** | ***D-A*** | ***P-A*** |
| NEO PI-R Facets |  |  |  |  |  |  |  |  |  |  |  |  |  |  |
| Anxiety | 59.26 | [54.53, 63.99] | 58.97 | [54.92, 63.02] | 59.62 | [57.10, 62.13] | 58.76 | [54.84, 62.67] | 0.03 | 0.03 | 0.04 | 0.06 | 0.02 | 0.08 |
| Angry Hostility | 69.27 | [64.50, 74.04] | 67.68 | [63.59, 71.76] | 62.02 | [59.49, 64.56] | 56.02 | [52.07, 59.96] | 0.14 | **0.64** | **1.16** | **0.50** | **1.02** | **0.53** |
| Depression | 63.52 | [58.65, 68.38] | 63.35 | [59.18, 67.52] | 60.87 | [58.28, 63.46] | 64.92 | [60.89, 68.94] | 0.01 | 0.23 | 0.12 | 0.21 | 0.13 | 0.35 |
| Self-consciousness | 66.11 | [61.22, 71.00] | 65.22 | [61.03, 69.40] | 60.56 | [57.96, 63.16] | 58.31 | [54.27, 62.36] | 0.08 | 0.48 | **0.67** | 0.40 | **0.59** | 0.19 |
| Impulsiveness | 65.24 | [59.56, 70.92] | 63.92 | [59.05, 68.79] | 61.60 | [58.57, 64.62] | 61.48 | [56.78, 66.19] | 0.10 | 0.27 | 0.28 | 0.17 | 0.18 | 0.01 |
| Vulnerability | 83.84 | [77.53, 90.16] | 87.58 | [82.17, 92.98] | 82.12 | [78.76, 85.48] | 58.88 | [53.66, 64.11] | 0.25 | 0.11 | **1.65** | 0.36 | **1.90** | **1.54** |
| Warmth | 44.63 | [38.88, 50.38] | 40.57 | [35.65, 45.49] | 43.07 | [40.02, 46.13] | 51.72 | [46.97, 56.47] | 0.30 | 0.11 | **0.52** | 0.18 | **0.81** | **0.63** |
| Gregariousness | 56.13 | [51.27, 61.00] | 51.19 | [47.02, 55.35] | 58.82 | [56.23, 61.41] | 49.52 | [45.49, 53.55] | 0.43 | 0.23 | **0.57** | **0.66** | 0.14 | **0.80** |
| Assertiveness | 61.12 | [56.30, 65.94] | 56.77 | [52.64, 60.89] | 57.06 | [54.50, 59.62] | 53.93 | [49.95, 57.92] | 0.38 | 0.35 | **0.62** | 0.03 | 0.25 | 0.27 |
| Activity | 54.95 | [50.24, 59.66] | 49.49 | [45.45, 53.52] | 52.44 | [49.94, 54.95] | 51.80 | [47.90, 55.70] | 0.49 | 0.22 | 0.28 | 0.26 | 0.21 | 0.06 |
| Excitement seeking | 48.45 | [43.77, 53.13] | 48.61 | [44.60, 52.62] | 49.86 | [47.37, 52.35] | 55.94 | [52.07, 59.81] | 0.01 | 0.13 | **0.67** | 0.11 | **0.65** | **0.54** |
| Positive emotions | 52.50 | [47.44, 57.57] | 45.44 | [41.11, 49.78] | 51.98 | [49.29, 54.67] | 49.86 | [45.67, 54.05] | 0.20 | 0.04 | 0.22 | **0.54** | 0.37 | 0.17 |
| Fantasy | 65.30 | [60.68, 69.92] | 65.87 | [61.91, 69.83] | 62.97 | [60.52, 65.43] | 57.28 | [53.46, 61.10] | 0.05 | 0.21 | **0.73** | 0.26 | **0.78** | **0.52** |
| Aesthetics | 58.02 | [53.83, 62.21] | 53.33 | [49.74, 56.92] | 55.58 | [53.36, 57.81] | 54.58 | [51.11, 58.05] | 0.47 | 0.24 | 0.34 | 0.23 | 0.12 | 0.10 |
| Feelings | 58.72 | [53.43, 64.01] | 53.49 | [48.96, 58.02] | 53.52 | [50.71, 56.33] | 53.63 | [49.25, 58.00] | 0.42 | 0.41 | 0.40 | 0.00 | 0.01 | 0.01 |
| Actions | 66.49 | [61.49, 71.48] | 65.89 | [61.62, 70.16] | 65.29 | [62.63, 67.94] | 54.48 | [50.35, 58.61] | 0.04 | 0.09 | **0.92** | 0.05 | **0.86** | **0.84** |
| Ideas | 55.69 | [51.23, 60.15] | 55.27 | [51.45, 59.09] | 51.62 | [49.25, 53.99] | 55.17 | [51.48, 58.86] | 0.04 | 0.38 | 0.05 | 0.34 | 0.01 | 0.33 |
| Values | 54.10 | [48.49, 59.71] | 53.28 | [48.48, 58.08] | 54.96 | [51.97, 57.94] | 58.29 | [53.65, 62.93] | 0.06 | 0.06 | 0.31 | 0.13 | 0.37 | 0.25 |
| Trust | 53.45 | [48.02, 58.87] | 49.18 | [44.54, 53.83] | 51.00 | [48.12, 53.88] | 46.55 | [42.06, 51.04] | 0.33 | 0.19 | **0.53** | 0.14 | 0.20 | 0.34 |
| Straightforwardness | 50.10 | [44.00, 56.19] | 51.65 | [46.43, 56.86] | 51.30 | [48.06, 54.54] | 45.48 | [40.44, 50.52] | 0.11 | 0.08 | 0.32 | 0.02 | 0.42 | 0.40 |
| Altruism | 47.39 | [41.87, 52.92] | 47.18 | [42.45, 51.91] | 47.09 | [44.16, 50.03] | 50.42 | [45.85, 54.99] | 0.02 | 0.02 | 0.23 | 0.01 | 0.25 | 0.25 |
| Compliance | 59.52 | [53.18, 65.86] | 58.24 | [52.81, 63.66] | 59.99 | [56.62, 63.36] | 43.37 | [38.13, 48.62] | 0.09 | 0.03 | **1.07** | 0.12 | **0.98** | **1.10** |
| Modesty | 56.44 | [50.99, 61.90] | 54.98 | [50.31, 59.65] | 55.50 | [52.60, 58.40] | 48.62 | [44.10, 53.13] | 0.11 | 0.07 | **0.60** | 0.04 | 0.49 | **0.53** |
| Tendermindedness | 61.00 | [55.60, 66.41] | 60.97 | [56.34, 65.59] | 57.29 | [54.42, 60.16] | 51.08 | [46.61, 55.55] | 0.00 | 0.29 | **0.77** | 0.29 | **0.77** | 0.48 |
| Competence | 51.14 | [45.22, 57.05] | 49.80 | [44.73, 54.86] | 49.40 | [46.26, 52.55] | 45.36 | [40.47, 50.26] | 0.10 | 0.12 | 0.41 | 0.03 | 0.31 | 0.29 |
| Order | 60.77 | [55.30, 66.25] | 59.26 | [54.57, 63.95] | 56.75 | [53.84, 59.67] | 45.24 | [40.71, 49.77] | 0.12 | 0.31 | **1.19** | 0.19 | **1.07** | **0.88** |
| Dutifulness | 47.12 | [41.92, 52.32] | 46.63 | [42.18, 51.09] | 47.24 | [44.48, 50.01] | 40.44 | [36.14, 44.74] | 0.04 | 0.01 | **0.54** | 0.05 | **0.50** | **0.55** |
| Achievement striving | 54.02 | [48.07, 59.97] | 49.91 | [44.82, 55.01] | 53.98 | [50.82, 57.15] | 45.09 | [40.16, 50.01] | 0.29 | 0.00 | **0.63** | 0.29 | 0.34 | **0.62** |
| Self-discipline | 52.91 | [47.42, 58.40] | 43.84 | [39.15, 48.54] | 50.62 | [47.70, 53.53] | 35.97 | [31.43, 40.51] | **0.70** | 0.18 | **1.29** | **0.52** | **0.60** | **1.12** |
| Deliberation | 63.25 | [58.02, 68.47] | 61.39 | [56.92, 65.86] | 59.43 | [56.65, 62.21] | 41.79 | [37.47, 46.11] | 0.15 | 0.31 | **1.72** | 0.16 | **1.57** | **1.41** |

Values represent the estimated marginal means and 95% confidence interval [lower bound, upper bound] with covariates of sex and age = 43.05 in the model.

^a^AUD – Alcohol use disorder

^b^Effect sizes are represented as, B-D –Bipolar to Depressive; B-P – Bipolar to Psychotic; B-A – Bipolar to AUD; D-P– Depressive to Psychotic; D-A- Depressive to AUD; P-A – Psychotic to AUD.

Effect sizes of medium magnitude (d = 0.5) or higher are **boldfaced**.

NEO-PI-R domain *T*-scores are presented.
