# Supplementary material for: Normative and Maladaptive Personality Trait Models of Mood, Psychotic, and Substance Use Disorders
Source: J Psychopathol Behav Assess. 2018 Jun 13;40(4):606–13. doi: 10.1007/s10862-018-9688-0 (PMC6223804; doi:10.1007/s10862-018-9688-0)

Supplementary Fig. 2: Effect sizes of the NEO PI-R facets, averaged and collapsed across pairwise comparisons

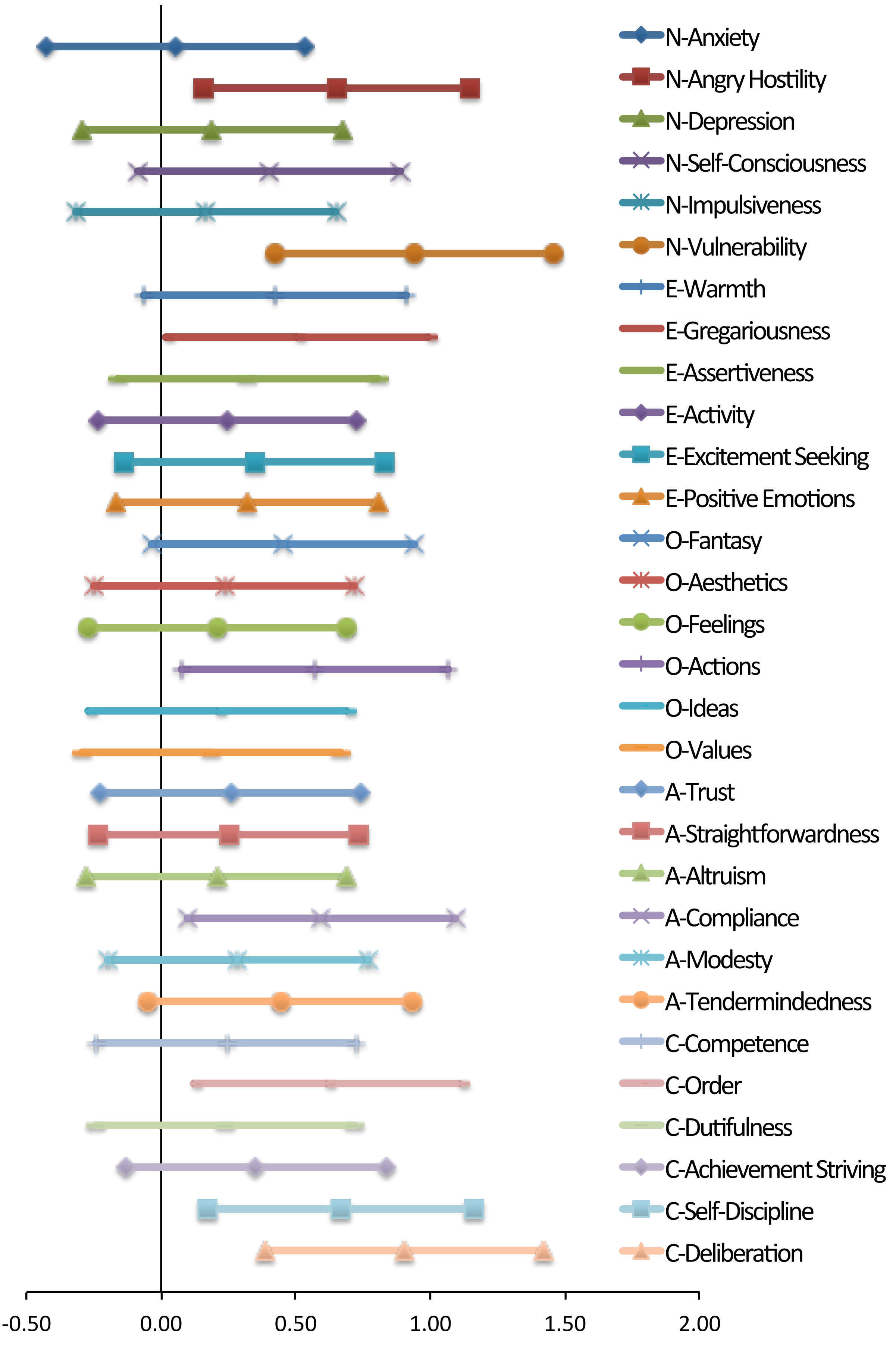

Supplement: Supplementary file 3 — (PDF 107 kb) [file 10862_2018_9688_MOESM3_ESM.pdf]
